# The Efficacy of Episil® for Radiation-induced Oral Mucositis in Rats

**DOI:** 10.7759/cureus.88479

**Published:** 2025-07-21

**Authors:** Shinichiro Kato, Hironori Miyamoto, Yosuke Naka, Koki Hayashi, Kenji Ohara, Kenichiro Ishibashi, Yasuyuki Shibuya

**Affiliations:** 1 Department of Oral and Maxillofacial Surgery, Nagoya City University West Medical Center, Nagoya, JPN; 2 Department of Oral and Maxillofacial Surgery, Nagoya City University Graduate School of Medical Sciences, Nagoya, JPN

**Keywords:** episil®, head and neck cancer, oral mucositis, radiation therapy, rats

## Abstract

Objective: Radiation therapy is an indispensable treatment for head and neck cancer; however, the development of radiation-induced oral mucositis causes erythema and ulcers in the patient's oral cavity, resulting in severe pain. The purpose of this study was to investigate the efficacy of Episil® in the treatment of radiation-induced oral mucositis.

Study design: Seventy-six F344 rats were randomly divided into an Episil® group (n=38) and a control group (n=38). After irradiation, the rats were evaluated based on body weight measurements, the oral mucositis index (OMI), tissue myeloperoxidase (MPO) activity, and intraoral bacteria counts.

Results: The mean ratio of body weight change in the Episil®group was significantly lower than that in the control group. The maximum mean OMI and maximum mean MPO activity did not differ to a statistically significant extent between the Episil®​​​​​​​ and control groups. The mean bacterial count in the Episil®​​​​​​​ group was significantly lower than that in the control group on days 8-12.

Conclusions: It is hypothesised that Episil®​​​​​​​ prevents bacterial infections and body weight loss, potentially suppressing the aggravation of oral mucositis. It is necessary to investigate the bacterial count, the bacterial environments, oral management and the severity of oral mucositis in clinical trials in future to verify the bacterial prevention effect of Episil®​​​​​​​.

## Introduction

Radiation therapy is an indispensable treatment for head and neck cancer, but approximately 95% of patients develop oral mucositis [1-3]. The development of oral mucositis causes erythema and ulcers in the oral cavity, resulting in severe pain. Severe oral mucositis can hinder conversation and oral intake and may be interrupted or discontinued during radiation therapy [4,5]. Preventing the development of severe oral mucositis is an important factor in radiation therapy, but no effective treatment has been established.

Episil® is a liquid device developed to manage and relieve pain in oral mucositis associated with cancer chemotherapy and/or radiotherapy. When applied to the oral mucosa, the water from the oral mucosa is absorbed, and gel is formed within a few minutes. External stimulation is then blocked, and pain is alleviated by the formation of a physical barrier [6]. In Japan, medical equipment manufacturing and sales approval was obtained in 2017, and Episil® received insurance coverage as a specific insurance medical material in 2018. Various studies have been conducted on the effect of pain, and it has generally been reported to have good analgesic effects [7-10].

We administered Episil® to patients who developed oral mucositis following head and neck radiation therapy. Clinically, we observed that the severity of mucositis appeared to be milder with Episil® use. Based on these observations, we hypothesised that Episil® may not only provide analgesic relief but also reduce the severity of oral mucositis. To verify this hypothesis, we conducted animal experiments to evaluate its potential therapeutic effect.

## Materials and methods

**Animals

**Figure 1:**
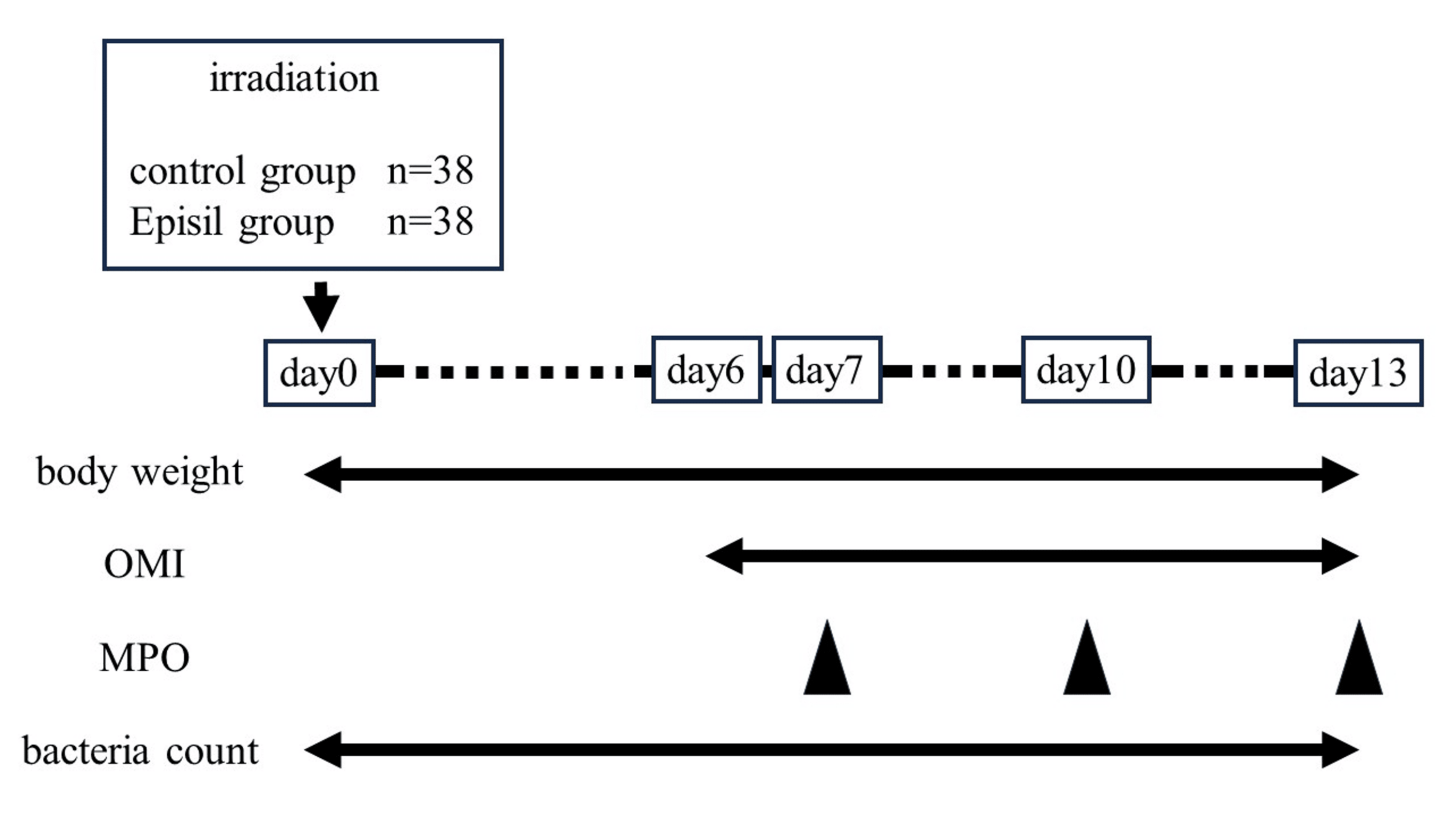
Seventy-six rats were randomly divided into an Episil® group (n=38) and a control group (n=38). After irradiation, the body weight and bacterial counts were measured from days 0 to 13, OMI from days 6 to 13, and MPO activity on days 7, 10, and 13.

Seventy-six, 8-week-old female Fisher 344 rats were purchased from Chubu Kagaku Shizai Co., Ltd. (Nagoya, Japan). The rats were kept in cages in animal rooms at the Center for Experimental Animal Science, Nagoya City University, where a controlled 12-hour light/12-hour dark cycle was maintained. The rats were fed a commercially available pellet diet (Chubu Kagaku Shizai Co., Ltd., Nagoya, Japan) and allowed ad libitum access to potable water. This study was conducted in accordance with the Nagoya City University Hospital Guidelines for Laboratory Animal Experiments and approved by the Experimental Animal Care Committee of this institution (H30-14) (Figure 1).

Irradiation

Irradiation was performed using a 210-kVp X-ray machine (10 mA with Al and Cu filters) as previously described [11]. The rats were randomly divided into an Episil® group (n=38) and a control group (n=38). Based on the results of previous research, we performed 18 Gy X-ray irradiation in each group, causing oral mucositis. Rezvani et al. demonstrated that radiation-induced oral mucositis occurred in 100% of rats exposed to 18 Gy and in 75% of those exposed to 16.5 Gy, based on their experiments involving graded radiation doses ranging from 13.5 to 18 Gy [12]. In 2015, we conducted a study using varying doses of X-ray irradiation on the tongues of F344 rats and confirmed that a dose of 18 Gy effectively induced oral mucositis [13]. In this study, the F344 rat tongue was irradiated by X-rays of 18Gy based on the previous study, and oral mucositis was caused. Oral mucositis was induced by a single dose of radiation via exposure of the right surface of the tongue, as it was pulled straight out of the mouth under general anesthesia using dexmedetomidine hydrochloride, midazolam, and butorphanol tartrate.

Application of Episil®

In the Episil® group, Episil® was applied to the tongue by the experimenter under general anesthesia once per day at a set time of 13 days after irradiation. The rats in the control group received general anesthesia once per day at a set time, 13 days after irradiation, but did not receive Episil®.

Body weight

Based on the results of our previous research, the rats in each group were individually weighed once a day at a set time on days 0-13 after irradiation. The amount of food being taken orally was not measured.

Oral mucositis index (OMI)

With the animal restrained, the tongue was gently pulled out from the oral cavity, and oral mucositis on the lingual dorsum of each rat was evaluated once daily from days 6 to 13 post-irradiation using a grading scale previously applied in similar experiments. Evaluations were performed by the same observer (Table 1) [14].

**Table 1:** Oral mucositis scoring system adapted from the system proposed by Parkins et al. [14] Used with permission from Parkins et al. [14].

Score	Description
0	Nomal
0.5	Slightly pink
1	Slightly red
2	Severely red
3	Focal desquamation
4	Exudate covering less than one-half of the irradiated mucosa
5	Virtually compete ulceration of the mucosa

Myeloperoxidase (MPO) activity

Rats in each group were sacrificed by an anesthetic overdose. Twenty-seven rats from each group were sacrificed 7, 10, and 13 days post-irradiation. The tongue was severed from its base and homogenised. The tongue homogenate was centrifuged at 4°C for 10 min at 13,000 g. The MPO in the resulting supernatant was activated with tetramethylbenzene and hydrogen peroxide solution, and spectrophotometric determination of the absorbance at 412 nm was then performed. MPO activity was determined using an MPO activity assay kit (Myeloperoxidase Coloring Activity Assay Kit®, Bio Vision Research Products, CA, USA) and MPO activity was expressed as the quantity of MPO per gram.

Intraoral bacterial count

Bacterial culture samples were collected once a day at a set time. A rapid bacterial quantification system (Bacteria Counter, DU-AA01NP-H; Panasonic Healthcare Co. Ltd., Tokyo, Japan) was used to measure the bacterial counts. This device can quantify data ranging from 1.0 × 10^5^ colony-forming units (CFU)/mL to 1.0 × 10^8^ CFU/mL.

Statistical analysis

Body Weight

Based on the results of the body change ratio in our previous research [11], we predicted a standard deviation of 12%, set the effect size at 6%, α error at 0.05, and power at 0.8, and required a sample of four rats per group.

OMI

Based on the results of our previous research [11], we predicted a standard deviation of 0.43, set the effect size at 0.4, α error at 0.05, and power at 0.8, and required a sample of 20 rats per group.

MPO Activity

Based on the results of our previous research [11], we predicted a standard deviation of 8 × 10^4^/g, set the effect size at 14 × 10^4^/g, α error at 0.05, and power at 0.8, and required a sample of six rats per group. MPO was measured on days 7, 10, and 13, requiring samples from 18 rats per group.

Intraoral Bacteria Count

Based on the research by Hayashi et al. [13], we predicted the standard deviation to be approximately 90 × 10^5^ CFU/mL, set the effect size to 130 × 10^5^ CFU/mL, the alpha error to 0.05, and the statistical power to 0.8, and required a sample of eight rats per group.

We estimated that a minimum sample of 76 rats (40 rats were measured for the body weight, OMI, and bacterial count, 36 rats were measured for MPO activity) would be needed.

Data Analysis

The data were expressed as the mean ± standard error. Significant differences were determined using a two-sided Student’s t-test. Statistical analyses were performed using SPSS (IBM Corp., 2019, IBM SPSS Statistics for Windows, Version 26.0. Armonk, NY: IBM Corp.), and statistical significance was set at *p<0.05.

## Results

Body weight

**Figure 2:**
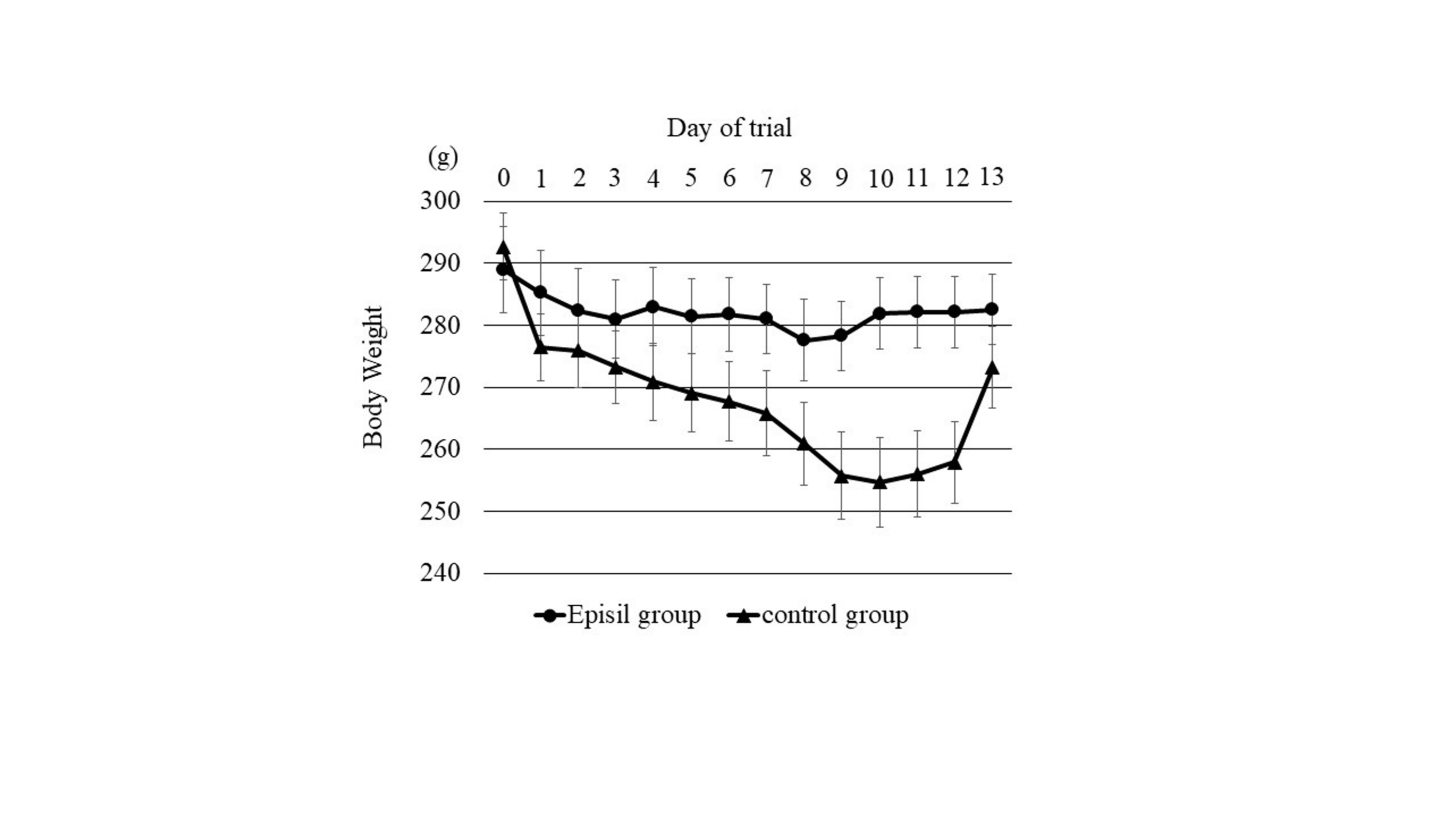
Rats in each group were weighed daily. The findings are expressed as changes in body weight relative to the initial body weight at the beginning of the experiment (day 0 of the trial) and represent the mean data for eight animals relative to the initial body weight values.

Each group of irradiated rats exhibited a reduction in body weight after irradiation. Thereafter, the decrease in body weight was maintained over days 1-3 post-irradiation, regardless of the dose of exposure. From day 4 post-irradiation onwards, the body weight of rats in the Episil® group showed no major changes, whereas the body weight of rats in the control group displayed substantial and persistent weight loss, which persisted for 10 days in the control group (Figure 2).

The maximum mean body weight loss value was −11.30 ± 7.35 g on day 8 post-irradiation in the Episil® group, whereas it was −35.80 ± 11.19 g on day 10 post-irradiation in the control group; the difference was statistically significant (Table 2).

**Table 2:** On day 1-13 post-irradiation, the mean body weights in the Episil® group was smaller than that in the control group.

Groups	Maximum weight loss value	The measurement date that recorded the maximum weight loss	t-score	p-value
Episil group	−11.30 ± 7.35 (g)	day 8	5.785	p<0.01
Control group	−35.80 ± 11.19 (g)	day 10		

Oral mucositis index (OMI)

**Figure 3:**
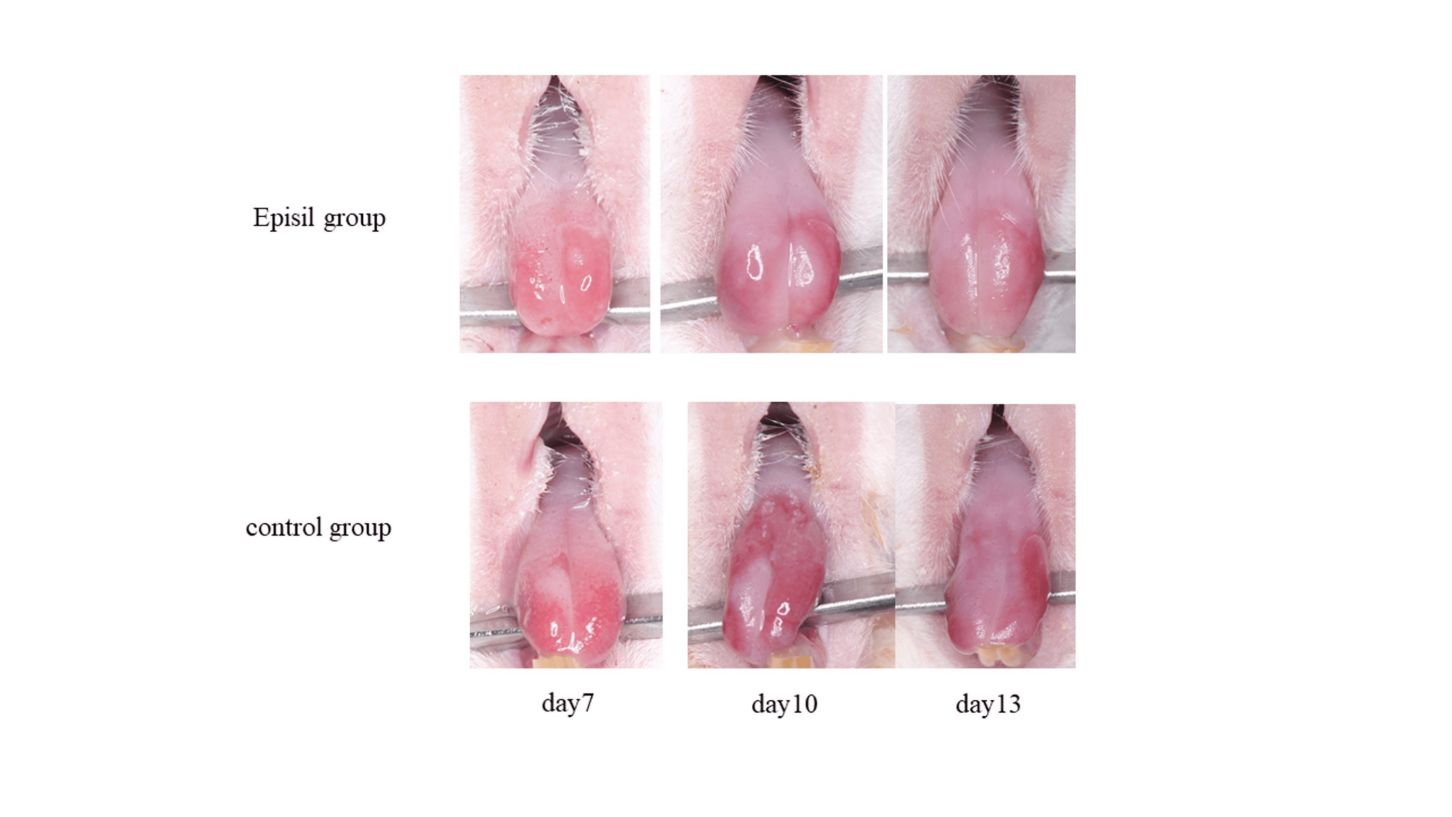
Macroscopic observation of the rat tongue on days 7, 10 and 13 post-irradiation.

On day 6 post-irradiation, lingual epithelial loss was observed in several rats in each group, and lingual epithelial loss was evident in all rats on day 7 post-irradiation. On day 10 post-irradiation, epithelialization on the tongues of the rats was observed in the Episil® group, although there were extensive epithelial defects on the tongues of the rats in the control group. On day 13 post-irradiation, epithelialization of the tongues of the rats was observed in each group (Figure 3).

**Figure 4:**
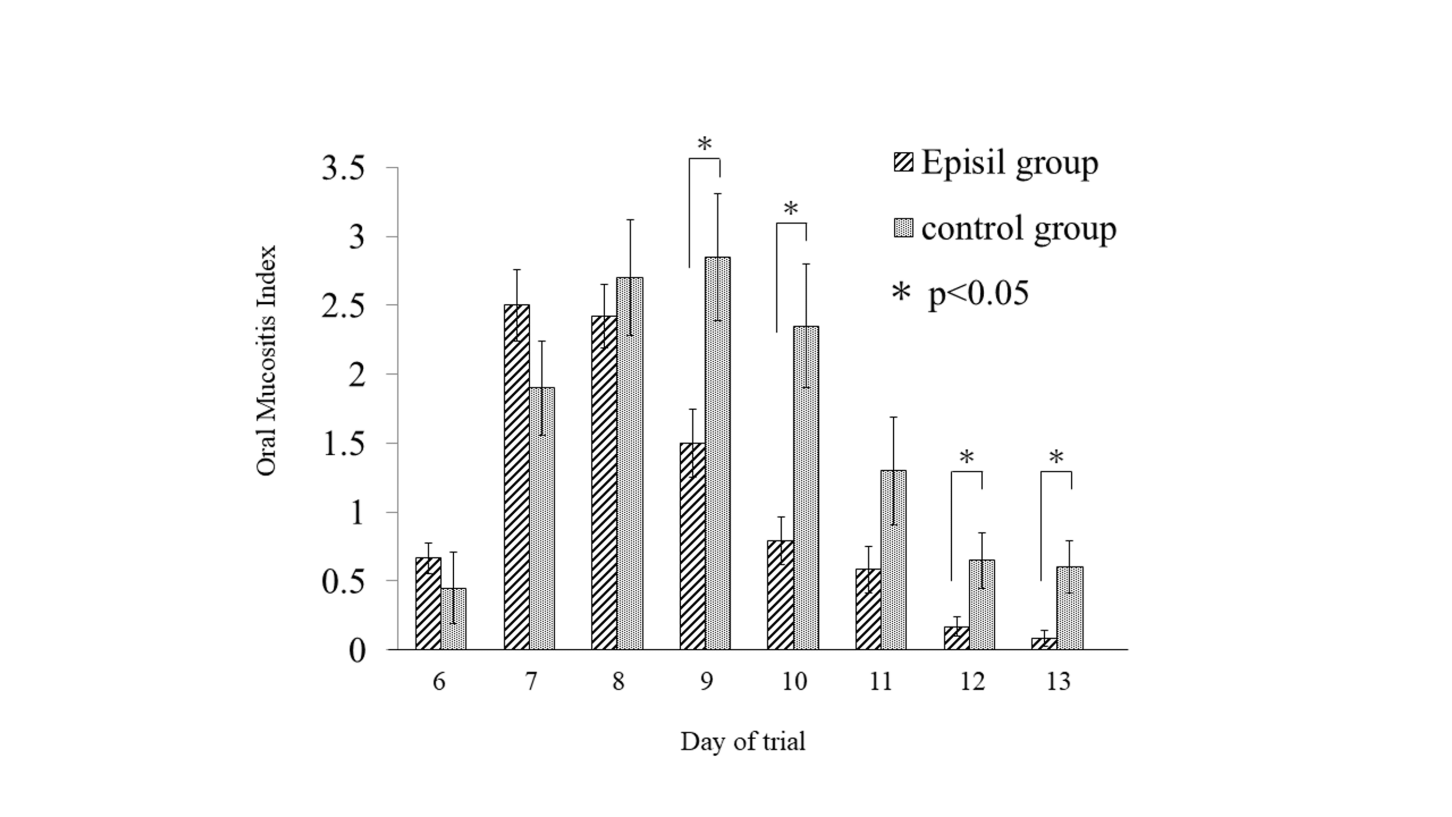
Once a day on days 6 to 13 post-irradiation, oral mucositis of the lingual dorsum of each rat was evaluated using a grading scale that has been used in previous experiments.

The mean OMI score on day 7 post-irradiation was 2.50 ± 0.26 in the Episil® group and 1.90 ± 0.34 in the control group; there were no significant differences in the mucositis scores between the groups. In the Episil® group, the mean OMI score was the highest on day 7 post-irradiation and then decreased. In the control group, the mean OMI score was the highest on day 9 post-irradiation and then decreased. The mean OMI score on day 9 post-irradiation was 1.50 ± 0.25 in the Episil® group and 2.85 ± 0.46 in the control group. On days 9, 10, 12, and 13 post-irradiation, the scores in the control group were significantly higher than those in the Episil® group (Figure 4). The most severe mean OMI score was 2.50 ± 0.26 on day 7 post-irradiation in the Episil® group and 2.85 ± 0.46 on day 9 post-irradiation in the control group. No significant differences were observed between the groups (Table 3).

**Table 3:** The most severe mean OMI in the Episil® and control groups did not differ to a statistically significant extent.

Groups	Most severe mean OMI	Number of days after irradiation	t-score	p-value
Episil group	2.50 ± 0.26	day 7	0.662	p>0.05
Control group	2.85 ± 0.46	day 9		

MPO activity

**Figure 5:**
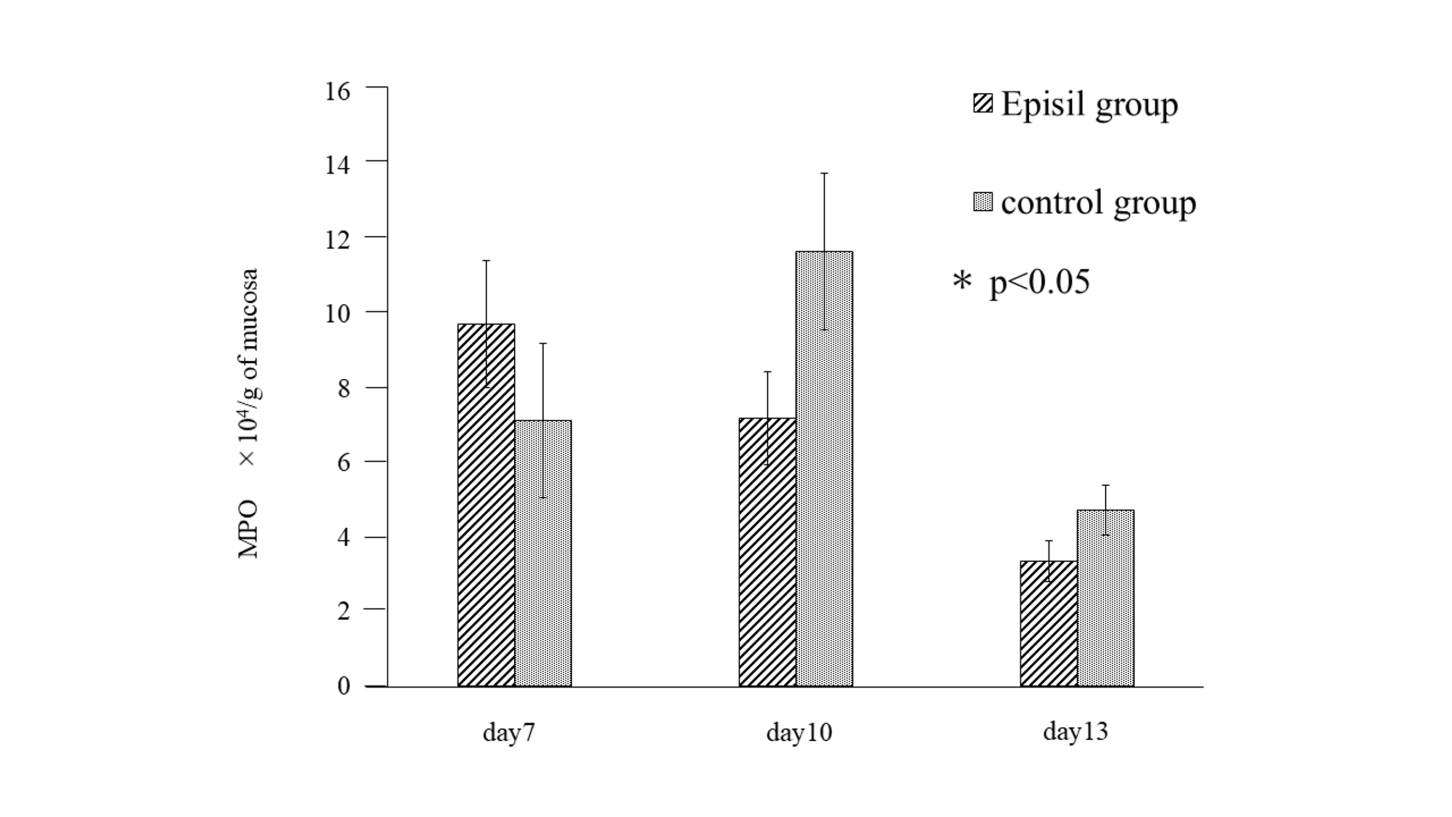
Tissue myeloperoxidase (MPO) activity on days 7, 10 and 13 post-irradiation.

The mean MPO activity on day 7 post-irradiation was 9.75 ± 1.71 × 10^4^/g in the Episil® group and 10.71 ± 2.07 × 10^4^/g in the control group. The mean MPO activity on day 10 post-irradiation was 7.22 ± 1.26 × 10^4^/g in the Episil® group and 11.70 ± 2.10 × 10^4^/g in the control group. The mean MPO activity on day 13 post-irradiation was 3.36 ± 0.55 ×10^4^/g in the Episil® group and 4.75 ± 0.67 × 10^4^/g in the control group. On all experimental days, there were no significant differences in MPO activity, although a slight non-significant difference was observed between the groups (Figure 5). There was no significant difference between the day 7 post-irradiation values of the Episil® group and the day 10 post-irradiation values of the control group (Table 4).

**Table 4:** The highest mean MPO activity in the Episil® and control groups did not differ to a statistically significant extent.

Groups	The highest mean MPO activity	Number of days after irradiation	t-score	p-value
Episil group	9.75 ± 1.71 (×10^4^/g)	day 7	0.722	p>0.05
Control group	11.70 ± 2.10 (×10^4^/g)	day 10		

Intraoral bacterial count

**Figure 6:**
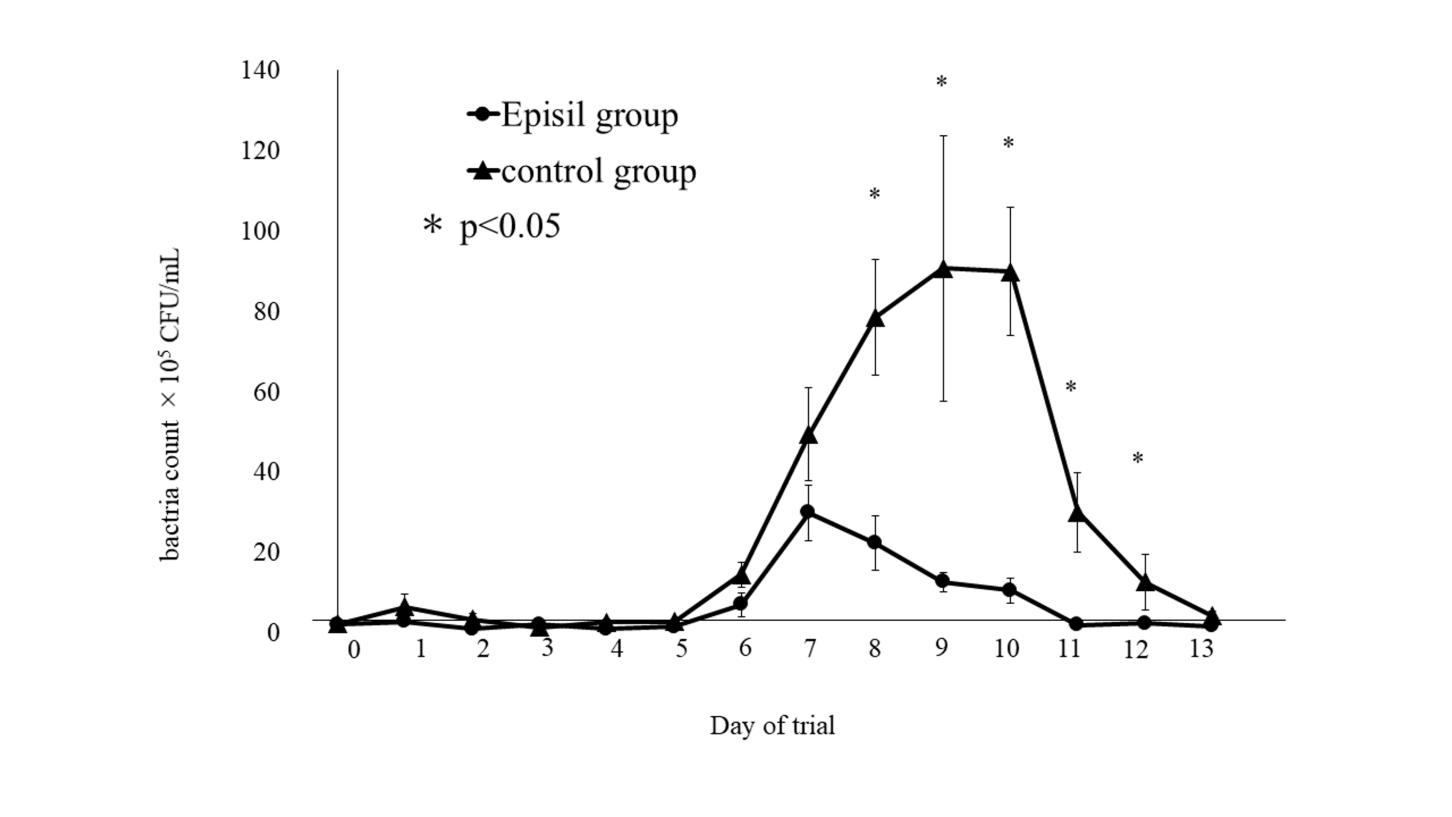
Bacterial culture samples were investigated once per day at a set time.

On day 0, the mean bacterial count in the Episil® group was 2.26 ± 0.49 × 10^5^ CFU/mL, the mean bacterial count in the control group was 2.41 ± 0.76 × 10^5^ CFU/mL, and there were no significant differences between the groups. The mean bacteria count has begun to increase in both groups from day 6, which developed oral mucositis. In the Episil® group, the mean bacterial count was recorded from day 6 post-irradiation, and the highest value was observed on day 7 post-irradiation (29.95 ± 6.91 × 10^5^ CFU/mL). The value decreased (A1) from day 8 post-irradiation. In the control group, as in the Episil® group, the mean bacterial count was recorded from day 6 post-irradiation, but the highest value was observed on day 10 post-irradiation (90.89 ± 33.04 × 10^5^ CFU/mL). The value decreased from day 11 post-irradiation. On days 8-12 post-irradiation, the mean bacterial count in the Episil® group was significantly lower than that in the control group (Figure 6). 

**Figure 7:**
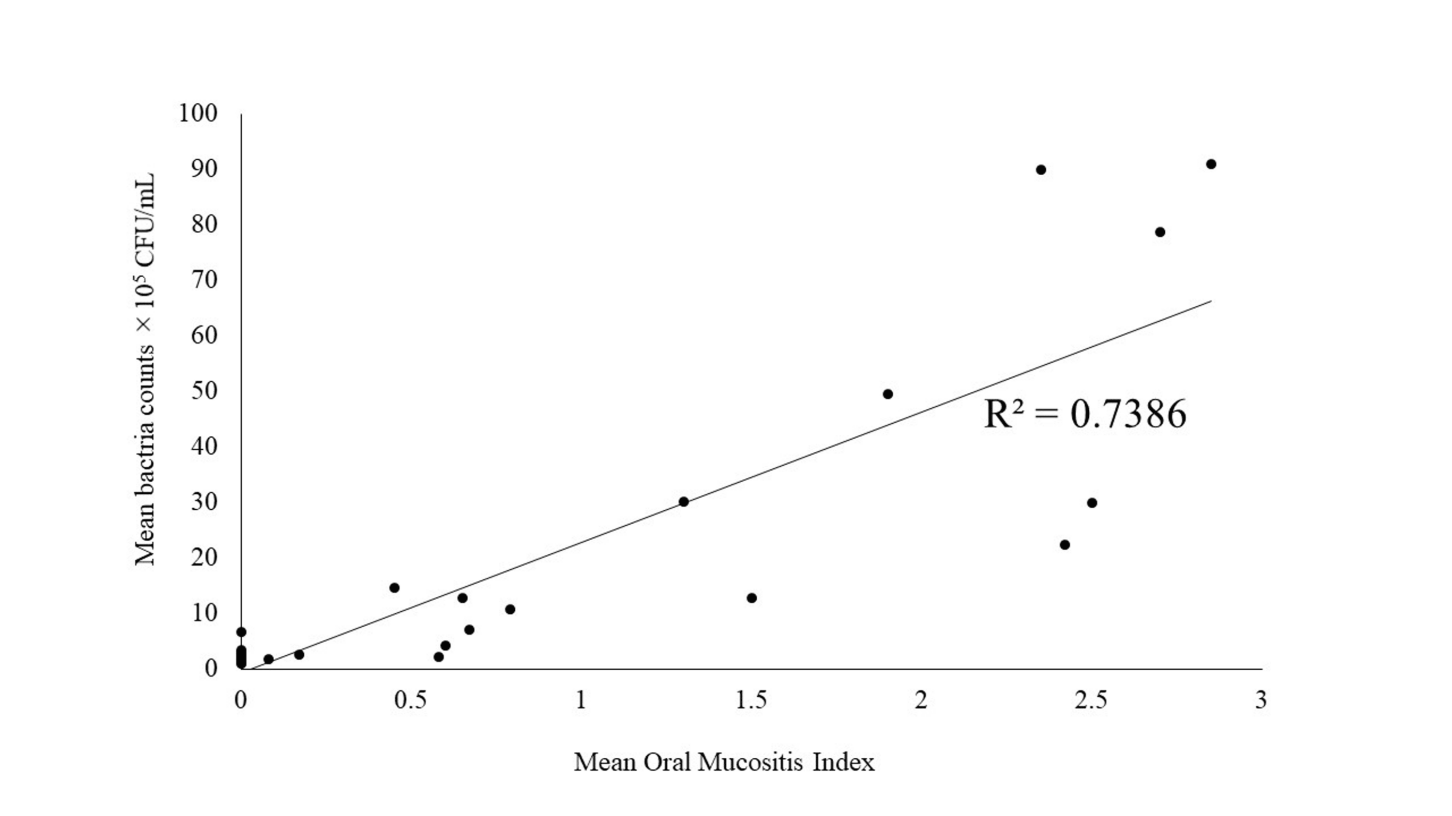
A correlation was found between the mean OMI and the mean bacteria count. Spearman’s rank correlation coefficient was 0.859.

 A correlation was found between the mean OMI and the mean bacteria count. Spearman’s rank correlation coefficient was 0.859 (Figure 7).

## Discussion

Various studies have been conducted on Episil®, but all of them have been on its analgesic effect on oral mucositis, and most of these studies have been clinical studies involving actual patients. The pain-relieving effect of Episil® has been demonstrated in many reports, but there has been little mention of its relationship with the severity of oral mucositis, as Episil® was originally developed as a bio-barrier material for pain management purposes [7-10].

In this study, no significant difference was observed in the severity of the most severe oral mucositis; however, the Episil® group had a shorter period of severe oral mucositis and tended to heal earlier. The first reason is that the nutritional status was better than that of the control group due to the analgesic effect of Episil®. Relative to the control group, weight loss was significantly lower, and the nutritional status was better in the Episil® group.

Nutritional status is an important factor in the healing of oral mucositis [15-17]. In 2021, Wei et al. reported that the incidence of grade 3-4 severe oral mucositis was significantly lower in 25 patients who received Episil® out of 50 patients with head and neck cancer who underwent radiation therapy compared to 25 patients who did not receive Episil® (p=0.037), and the reason for this was reported to be good nutritional status [18].

On the other hand, there are reports that the use of Episil® can prevent the development of severe radiation dermatitis, not just radiation oral mucositis, which is related to the nutritional status, so it is possible that factors other than the nutritional status are involved in the worsening of inflammation. In 2024, Li et al. reported that among 100 lung cancer patients undergoing radiation therapy, 65 patients used Episil® as a protective material on the skin, and the incidence of severe dermatitis was significantly lower than that in 35 patients in whom Episil® was not used [19]. They speculated that this result was due to the fact that applying Episil® reduced the effects of skin reactions, such as pain and itching.

A second possible reason why the use of Episil® led to earlier healing of oral mucositis is that it prevented bacterial infection. In this study, no significant differences were observed between the groups in OMI and MPO measurements at the most severe stage. However, the results showed that the severity of oral mucositis and the bacterial count in the oral mucositis ulcers of the Episil® group were significantly lower than that of the control group, and a correlation was found between the mean OMI and the mean bacterial count. The correlation between the mean OMI and the mean bacterial count was considered to be associated with the severity of oral mucositis and the bacterial counts. It is not clear whether severe oral mucositis causes the increase in bacteria or the increase in bacteria has led to the severity of oral mucositis in this study. However, based on previous reports, we consider that bacterial infection in the ulcerative site may have led to the aggravation of oral mucositis [20-22]. Cell wall products from incubating bacteria travel through the disrupted epithelium into the submucosa, where they stimulate macrophages to produce additional proinflammatory cytokines [23]. It is thought that the physical defensive effect of Episil® suppresses secondary bacterial infection, allowing mucosal healing to begin earlier.

A third reason why the use of Episil® led to earlier healing of oral mucositis is that the soy-derived ingredients contained in Episil® may have reduced oral mucositis. The main ingredients of Episil® are glycerin dioleate and soy phosphatidylcholine (PC) [24,25]. PC is a lecithin with antioxidant effects. It is possible that lecithin was applied to ulcers for a long period and promoted tissue regeneration after absorption into tissue [26,27]. It is possible that the phosphatidylcholine contained in Episil® has an effect on the healing of oral mucositis, but it is necessary to verify whether it is absorbed into the tissue when administered locally. Among the above three factors, we considered that the effect of preventing bacterial infection by the barrier function of Episil® is the most involved in the healing of radiation oral mucositis. It is reported that the nutritional status and the severity of oral mucositis in radiotherapy for head and neck cancer were not related. And in the same study, it is reported that the oral bacteria and the severity of oral mucositis in the radiotherapy for head and neck cancer were related.

Episil® has previously been recognised solely for its physical defence-based analgesic effects, but recent research suggests that it may also serve to alleviate the exacerbation of oral mucositis [18]. It has been suggested that the use of Episil® for mild oral mucositis, which does not require pain control, may prevent secondary bacterial infection and aggravate mucositis.

This study had several limitations. It is unknown whether the increased bacterial counts have prolonged the oral mucositis, or whether the prolonged oral mucositis development has increased the bacterial counts. Only the bacterial count is performed, and bacterial environments have not been identified in this study. In addition, this study did not measure the amount of food being taken orally. The decrease in the amount of food being taken orally may reduce the self-cleaning effect and increase bacteria. Rats and humans have different oral environments in terms of oral care and management, so it is necessary to carefully interpret that Episil® can be used to prevent bacterial infection in humans.

## Conclusions

It is hypothesised that Episil® prevents bacterial infections and body weight loss, potentially suppressing the aggravation of oral mucositis. It is necessary to investigate the bacterial count, the bacterial environment, oral management and the severity of oral mucositis in clinical trials in future to verify the bacterial prevention effect of Episil®.
